# SOCIAL SUPPORT USING SOCIAL NETWORKING SERVICES FOR OLDER ADULTS WITH MCI

**DOI:** 10.1093/geroni/igae098.4079

**Published:** 2024-12-31

**Authors:** Seolah Yoon, Jeongeun Choi, Min Kyung Park, Dahye Hong, Bada Kang

**Affiliations:** Yonsei University, Seoul, Republic of Korea; Yonsei University, Seoul, Republic of Korea; Yonsei University College of Nursing, Seoul, Republic of Korea; Yonsei University, Seoul, Republic of Korea; Yonsei University College of Nursing, Seoul, Republic of Korea

## Abstract

Older adults with mild cognitive impairment (MCI) are more likely to experience negative health-related outcomes. Social support is an important strategy for preventing adverse outcomes. While social support interventions using social networking services (SNSs) have potential benefits for social relationships and health, their impact on older adults with MCI, along with usability challenges and strategies, has not been comprehensively synthesized. The aim of this scoping review was to map the use of social support interventions using SNSs for health and social care of older adults with MCI and examine the usability challenges and strategies of SNS platforms. Following the Joanna Briggs Institute methodology, eight studies published between January 2005 and June 2024 were selected from such databases as PubMed, CINAHL, Web of Science, EMBASE, PsycINFO, SCOPUS, and Google Scholar. Social support interventions were categorized into five types: social networking, informational, esteem, emotional, and tangible support. Positive outcomes of the interventions included enhanced social interaction, increased physical activity, and improved memory and alertness. Usability challenges, such as technical skill deficits and privacy concerns, were identified, and strategies, such as weekly email checklists, training sessions, and simplified design features, have been proposed to address them. This review highlights the potential of social support interventions using SNSs to positively impact the physical, cognitive, and social outcomes of older adults with MCI. We discuss gaps in social support interventions attributed to SNSs and implications for future interventions aimed at improving the health and social well-being of older adults with MCI.

